# Production of single cell oil by *Yarrowia lipolytica* JCM 2320 using detoxified desiccated coconut residue hydrolysate

**DOI:** 10.7717/peerj.12833

**Published:** 2022-03-01

**Authors:** Muhammad Fakhri Zainuddin, Chong Kar Fai, Mohd Shamzi Mohamed, Nor ’Aini Abdul Rahman, Murni Halim

**Affiliations:** 1 Department of Bioprocess Technology, Faculty of Biotechnology and Biomolecular Sciences, Universiti Putra Malaysia, Serdang, Selangor, Malaysia; 2 Bioprocessing and Biomanufacturing Research Complex, Faculty of Biotechnology and Biomolecular Sciences, Universiti Putra Malaysia, Serdang, Selangor, Malaysia

**Keywords:** Lipid, Single cell oil, Desiccated coconut hydrolysate, Fermentation inhibitor, Detoxification, Adsorption resin, *Yarrowia lipolytica*, Oleaginous yeast, Overliming detoxification

## Abstract

Nowadays, the replacement of petro-diesel with biodiesel has raised the concern among the community for the utilization of improper feedstocks and the cost involved. However, these issues can be solved by producing single cell oil (SCO) from lignocellulosic biomass hydrolysates by oleaginous microorganisms. This study introduced *Yarrowia lipolytica* JCM 2320 with a desiccated coconut residue (DCR) hydrolysate (obtained from the 2% dilute sulphuric acid pretreatment) as a carbon source in generating SCO. However, common inhibitors formed during acid pretreatment of biomass such as five-hydroxymethylfurfural (HMF), furfural, acetic acid and levulinic acid resulting from the sugar degradations may have detrimental effects towards the fermentation process. To visualize the effect of inhibitors on *Y. lipolytica*, an inhibitory study was conducted by adding 0.5–5.0 g/L of potential inhibitors to the YPD (yeast, peptone and D-glucose) medium. It was found that the presence of furfural at 0.5 g/L would increase the lag phase, which beyond that was detrimental to *Y. lipolytica*. Furthermore, increasing the five-hydroxymethylfurfural (HMF) concentration would increase the lag phase of *Y. lipolytica*, whereas, for acetic acid and levulinic acid, it showed a negligible effect. Detoxification was hence conducted to remove the potential inhibitors from the DCR hydrolysate prior its utilization in the fermentation. To examine the possibility of using adsorption resins for the detoxification of DCR hydrolysate, five different resins were tested (Amberlite® XAD-4, Amberlite® XAD-7, Amberlite® IR 120, Amberlite® IRA 96 and Amberlite® IRA 402) with five different concentrations of 1%, 3%, 5%, 10% and 15% (w/v), respectively. At resin concentration of 10%, Amberlite® XAD-4 recorded the highest SCO yield, 2.90 ± 0.02 g/L, whereas the control and the conventional overliming detoxification method, recorded only 1.29 ± 0.01 g/L and 1.27 ± 0.02 g/L SCO accumulation, respectively. Moreover, the fatty acid profile of the oil produced was rich in oleic acid (33.60%), linoleic acid (9.90%), and palmitic acid (14.90%), which indicates the potential as a good biodiesel raw material.

## Introduction

Non-renewable fossil fuels, petroleum has been largely exploited over the past years for petro-diesel production to fulfil the energy demand of the world population (Das et al., 2017). In addition, its continual consumption would eventually lead to environmental pollutions such as global warming and air pollution (Singh, Suhag & Dhaka, 2015). With these challenges, petro-diesel has been shifted to a new emerging industry, namely, renewable, and eco-friendly biodiesel. However, the feedstock utilized in the biodiesel production involves a high cost (Sahar et al., 2018).

As an alternative, single cell oil (SCO), composed of fatty acid similar to conventional vegetable oils, is suggested to replace edible oils in producing biodiesel (Mhlongo et al., 2021; Spagnuolo, Yaguchi & Blenner, 2019). It can be obtained easily through the lipid fermentation process of oleaginous microorganisms with sufficient nutrients (Caporusso, Capece & De Bari, 2021). Lipids accumulation is initiated when there is inadequate nitrogen in the growth media, but with an excessive carbon source which will then be induced to metabolize into lipids in a series of chemical pathways (Liu Patel et al., 2020). Oleaginous microorganisms such as *Trichosporon fermentans* (yeast) (Liu et al., 2015), *Rhodococcus opacus* (bacteria) (Wei et al., 2015), *Mortierella isabelline* (fungus) (Yu et al., 2015) and *Chlorella sorokiniana* (microalgae) (Yu et al., 2015) are the key element in this process, and they have been proven to accumulate SCO of more than 20% of the biomass (Diwan, Parkhey & Gupta, 2018). As reported, oleaginous yeast is much preferable for producing SCO compared to other oleaginous microorganisms such as bacteria, fungi, or microalgae (Wu et al., 2018). For example, a non-pathogenic oleaginous yeast, *Yarrowia lipolytica*, has been widely applied for SCO production due to its complete genome (Nicaud, 2012).

Aiming to accumulate SCO in a more economically feasible way, researchers proposed replacing the costly synthetic glucose with lignocellulosic biomass, which is cheaper and abundant (Ram, Tyagi & Drogui, 2018; Valdés, Mendonça & Aggelis, 2020). Various lignocellulosic biomass has been explored as the fermentation substrates, such as barley straw (Kucharska et al., 2018), corn fibre spruce and wheat straw (Kim, 2018). Focusing on the industrial crops planted in Malaysia, coconut is ranked fourth after oil palm, rubber, and paddy in terms of the amount of plantation (Tan, 2019). Various value-added products emerge from the coconut fruit as well as its by-products. One of the famous examples of its by-products is desiccated coconut residue. It is white in appearance and is derived by shredding the dehydrated coconut meat (Rethinam, 2019). Desiccated coconut is one of the agricultural wastes that can be utilized as a low-cost feedstock which is believed to be a potential carbon source for microbial growth (Mohd Zin et al., 2017).

One of the main obstacles to producing second-generation biodiesel is improving the use of waste, especially agricultural materials as nutrients, to allow microorganisms to convert them into SCO fully. Lignocellulosic biomass is usually characterized by its high crystallinity and recalcitrant structure in the presence of structural components such as cellulose and lignin (Zoghlami & Paës, 2019). Therefore, the pretreatment process is utterly crucial to overcome this issue and enhance the hydrolysis of cellulose and hemicellulose into sugar monomers, which will be utilized by fermenting microorganisms as carbon sources (Kim, Lee & Kim, 2016). To date, several pretreatment methods have been explored, and they are mainly grouped according to their mode of action, for instance, physical, chemical, biological, and physicochemical pretreatments (Diwan, Parkhey & Gupta, 2018*; Zainuddin et al., 2021*). Inhibitory compounds, mainly obtained from the degradation of carbohydrates (such as pentoses and hexoses) and lignin polymer during the pretreatment of lignocellulosic biomass, will exhibit toxicity effect on fermenting microorganisms. Hence, cell growth and lipid accumulation are prohibited (Moreno et al., 2014). Common fermentation inhibitors include two-furaldehyde (furfural), five-hydroxymethylfurfural (HMF), acetic acid, levulinic acid, formic acid, and phenolic compounds (Paes et al., 2021). A detoxification step is thus necessary to remove the inhibitors from the hydrolysate (Sjulander & Kikas, 2020; Hong et al., 2021). Though it can be completed with different approaches, overliming and resin adsorption detoxification methods are most frequently implemented. To be more specific, anion exchange resins are receiving the greatest attention as it leads to the elimination of all types of inhibitors (Wang et al., 2020). Nevertheless, a variation in the selection of oleaginous microorganisms and their strains possess different tolerance towards inhibitors present in the hydrolysate (Almeida et al., 2007; Vanmarcke et al., 2021). An inhibitory study is hence essential to be completed prior to the detoxification method to determine the tolerance of the strain toward commonly found inhibitors and, in turn, selecting the optimal approaches for the greatest efficiency (Diwan, Parkhey & Gupta, 2018*; Sagia et al., 2020*). Thus, the main objective of this work was to investigate the capability of *Y. lipolytica* JCM 2320 in producing SCO in non-detoxified and detoxified desiccated coconut residue hydrolysates. The possibility of using ion exchange and polymeric resins for detoxification of desiccated coconut residue hydrolysates as an alternative to conventional overliming detoxification method was examined.

## Materials and Methods

### Yeast strain and growth medium

*Yarrowia lipolytica* JCM 2320 (Wickerham et al.) van der Walt & von Arx, simplified as *Y. lypolytica* JCM 2320 employed throughout this experimental study, was obtained from RIKEN BioResource Research Center, Japan. It was grown and maintained on a nutrient agar plate (10 g/L yeast extract, 20 g/L peptone and glucose, respectively, and 15 g/L agar) (Kim et al., 2013). Prior to fermentation, *Y. lipolytica* JCM 2320 was inoculated in YPD broth composed of 10 g/L yeast extract, 20 g/L peptone and 20 g/L D-glucose (Gong et al., 2014). It was then incubated in an incubated shaker (Lab Companion IS-971 R Floor Model) at 150 rpm, 30 °C for 24 h as a pre-culturing step. To initiate the fermentation process, 10% (v/v) of the seed inoculum was added to the fermentation media (Yu et al., 2015).

### Preparation of desiccated coconut residue

Desiccated coconut residue used throughout the whole experimental work was obtained from a local wet market. It was then dried in a dryer for 24 h at 60 °C to remove its moisture content. The moisture content was measured before and after 24 h of drying until the constant weight were obtained. Dried desiccated coconut residue was then sieved through a 1–2 mm sieve to remove large particles and was packed in plastic bags before storing at room temperature (Aboagye et al., 2017).

### Optimization of pretreatment condition

For acid hydrolysis, 10 g of dried desiccated coconut residue was treated with 100 mL dilute sulphuric acid (H_2_SO_4_) solution at concentrations of 0.5%, 1.0%, 2.0%, 3.0%, 5.0%, 6.0%, 8.0% and 10.0%. It was then autoclaved at 121 °C for 20 min and cooled down (Yu et al., 2011). The hydrolysate was obtained by vacuum filtration and was analyzed for its concentration of reducing sugar. While for alkaline hydrolysis, the procedure was repeated at the same concentrations by replacing the dilute H_2_SO_4_ with dilute sodium hydroxide (NaOH) solution (Aboagye et al., 2017).

### Screening of yeast tolerance to fermentation inhibitor compounds

Four different types of commonly reported fermentation inhibitors present in lignocellulosic biomass hydrolysates, namely HMF, furfural, acetic acid and levulinic acid, were added at various concentrations (0.5, 1.0, 2.0, 3.0, 4.0 and 5.0 g/L) to the YPD media. The media were then adjusted to a pH range of 5.5 to 6.5 with NaOH by pH meter (Eutech pH 700) before being autoclaved at 121 °C for 20 min. Once the media had completely cooled down, 10% (v/v) of the seed inoculum was added to the fermentation media to initiate the fermentation process. To determine the effect of inhibitors, a control was set up by inoculating *Y. lipolytica* JCM 2320 in YPD media without the addition of any inhibitors (Kim et al., 2013).

### Preparation of medium with undetoxified desiccated coconut residue hydrolysate

Desiccated coconut residue hydrolysate was used as an alternative source to the synthesized glucose. Fermentation medium composed of desiccated coconut residue hydrolysate with the concentration of 25 g/L (Medium 25), 20 g/L (Medium 20), 15 g/L (Medium 15), 10 g/L (Medium 10) and 5 g/L (Medium 5) reducing sugar were prepared together with 10 g/L yeast extract and 20 g/L peptone. Standards YPD medium composed of 10 g/L yeast extract, 20 g/L peptone, 20 g/L D-glucose (Medium YPD) and YP medium composed of 10 g/L yeast extract, 20 g/L peptone (Medium YP) were used as control experiments (Gong et al., 2014). 10% (v/v) of seed inoculum in the range of 0.6 to 0.8 optical density of 600 nm (OD_600_) was added to initiate the fermentation process (Yu et al., 2015). The fermentation process was performed in triplicate using three separate Erlenmeyer flasks (250 mL) in an incubated shaker with controlled parameters (30 °C and 150 rpm). To determine the best fermentation media to be used in the subsequent experiments, sampling was done at 24 h intervals for analyses such as cell growth, reducing sugar and yield of SCO.

### Detoxification of desiccated coconut residue by overliming method

In an overliming method, the media composed of desiccated coconut residue hydrolysate was stirred and heated on a heater stir plate (Thermolyne Cimarec 2 Hotplate stirrer). It was followed by the addition of calcium hydroxide powder (Ca (OH)_2_), until the pH value reaching 10, and using vacuum filtration, the mixture was then filtered to remove the precipitation. The pH of the filtered hydrolysate was then adjusted back to pH 5.5. It was then used as a fermentation substrate with yeast extract and peptone (Yu et al., 2011). Fermentation substrate with the addition of undetoxified hydrolysate was used as an experimental control.

### Resin properties and preparation

Five different types of resins were studied, these include Amberlite® IR 120 Hydrogen form, Amberlite® IRA 96 Free base, Amberlite® IRA 402 Chloride form, Amberlite® XAD-7 and Amberlite® XAD-4. All of them were purchased from Sigma Aldrich (Darmstadt, Germany). Polymeric adsorbent types (Amberlite® XAD-7 and Amberlite® XAD-4) were washed 3 times with two-propanol at a ratio of resins to solvent of 1:4 (w/v). After washing the resins, the mixture was separated by filtrating it with Whatman No.1 filter paper. The wet resins were then left until completely dried in an oven (60 °C) for further usage (Sandhya et al., 2013). For weak and strong anion exchange resins (Amberlite® IRA 96 free base and Amberlite® IRA 402), hydrochloric acid (HCl) and sodium hydroxide (NaOH) that were introduced in the washing procedure were responsible for removing the chemical residue on the resins and in turn, regenerated the reproducibility of the resins (Kocaoba, 2003). The resins were washed until final pH 7 according to the well-published sequences: 1N HCl solution, distilled water, 1 N NaOH solution, distilled water, 1 N HCl, and distilled water. Following that, they were left dried in a dryer for 24 h at 105 °C (John, Nampoothiri & Pandey, 2008). The cation exchange resin (Amberlite® IR 120) can be obtained in its H^+^ form through washing with 1 N HCl and was continued with distilled water (Bishai et al., 2015). Dried resins at initial concentrations of 1%, 3%, 5%, 10% and 15% (w/v) were then introduced into the sulphuric acid-pretreated desiccated coconut residue hydrolysate. The mixture was then detoxified by stirring on a stirrer plate for 1 h, and it was then filtered with vacuum filtration. The detoxified hydrolysate was then used as a fermentation substrate with the addition of yeast extract and peptone, and with pH adjustment to 5.

## Analytical methods

### Determination of cell growth

Cell growth of the samples from different sets of experiments were obtained by measuring OD at 600 nm with a UV-Vis spectrophotometer (Secomam UviLine 9400) (Fei et al., 2016). The growth curve of *Y. lipolytica* JCM 2020 was then plotted to show its growth profile.

### Determination of reducing sugar

The reducing sugar concentration was determined by 3,5-dinitrosalicylic acid (DNS) method (Gong et al., 2014). A standard curve was prepared before the DNS analysis. For DNS analysis, the aforementioned procedure was repeated by replacing the glucose with the samples obtained from the hydrolysate. The concentration of reducing sugar was then calculated according to the equation of the standard curve (Gusakov, Kondratyeva & Sinitsyn, 2011).

### Determination of cell dry weight

Cell dry weight measurement was initiated by centrifuging the samples obtained at 10,000 × *g* for 5 min. The cell pellet resulted from the centrifugation was then washed twice with distilled water. The sample was left dry in a dryer, and the cell dry weight was then determined gravimetrically (Maina et al., 2017). Cell dry weight was calculated by using Eq. (1):

(1) $${\rm Cell\; dry\; weight\; (g/L)} =\displaystyle{{Weight\; of\; the\; dried\; cell\; \left( g \right)} \over {Volume\; of\; biomass\; \left( L \right)}}$$

### Determination of single cell oil (SCO)

After cell dry weight determination, the dried yeast cell was then disrupted with pestle and mortar and extracted for SCO according to Bligh and Dyer method (Bligh & Dyer, 1959) with a slight modification as described by Vasconcelos et al. (2018). The SCO was analyzed according to Eqs. (2) and (3).

(2) $$\eqalign{
  & {\rm{Single}}\;{\rm{cell}}\;{\rm{oil}}\;{\rm{yield}}\;({\rm{g/L}})  \cr 
  &  = {{Weight\;of\;eppendorf\;tube\;with\;SCO\;\left( g \right) - Weight\;of\;empty\;eppendorf\;tube\left( g \right)} \over {Volume\;of\;sample\;\left( L \right)}} \cr} $$

(3) $${\rm Lipid \;content \;(\%)}=\displaystyle{{Single\; cell\; oil\; yield\; \left( {\displaystyle{g \over L}} \right)} \over {Cell\; dry\; weight\; \left( {\displaystyle{g \over L}} \right)}}\; x\; 100\%$$

### Determination of kinetic parameters

Cell dry weight yield coefficient per substrate (Y_X/S_), SCO yield per dry cell weight (Y_P/X_) as well as SCO yield coefficient per substrate (Y_P/S_) were calculated and tabulated according to Eqs. (4)–(6).

(4) $${\rm Cell \;dry \;weight \;yield \;coefficient \;per \;substrate \; (Y_{X/S})} =\displaystyle{{\; X - Xo} \over {So - S}}$$

where,

X = Maximum cell dry weight.

X_o_ = Initial cell dry weight.

S_o_ = Initial substrate.

S = Maximum substrate.

(5) $${\rm SCO \; yield \; coefficient \; per \; cell \; dry \; weight \; (Y_{P/X})} =\displaystyle{{\; P - Po} \over {X - Xo}}$$

where,

P = Maximum SCO yield.

P_o_ = Initial SCO yield.

X = Maximum cell dry weight.

X_o_ = Initial cell dry weight.

(6) $${\rm SCO \; yield \; coefficient \; per \; substrate \; (Y_{P/s}) =}\displaystyle{{\; P - Po} \over {So - S}}$$

where,

P = Maximum SCO yield.

P_o_ = Initial SCO yield.

S_o_ = Initial substrate concentration.

S = Maximum substrate concentration.

### Fatty acid determination

Fatty acids profile was determined as the fatty acid methyl esters (FAME) by gas chromatography (Harmanescu, 2012). The yeast cells were collected and freeze-dried overnight. The fatty acid composition of the FAME was extracted by diethyl ether and petroleum ether. Then, the methylation process was conducted by adding 7% boron trifluoride in methanol solution and toluene into a flask containing oil or fat extracts. Ultrapure water, heptane and Na_2_SO_4_ were added to extract FAME in the top layer formed. The top layer was then transferred into a vial and injected into a gas chromatography flame ionization detector (Shimadzu GC-17A) inlet for analysis. The GC was conducted using capillary column Rt-2560 (100 m × 0.25 mm ID × 0.20 µm film) (from Restek Corporation, Bellefonte, PA, USA) with flow rate helium (0.75 mL/min), hydrogen (45 mL/min) and air (450 mL/min). The injector temperature was at 225 °C, and the detector temperature was at 285 °C. The initial temperature was set up at 100 ˚C and held for 4 min, increased by 3 °C/min to 240 °C and then hold at this temperature for 15 min. Fatty acid components are reported as percent (%) based on the percentage area of each fatty acid peak. Total peak area was calculated as the sum of all identified peaks of FA (Savchenko et al., 2021).

### Statistical analysis

**Figure 1:**
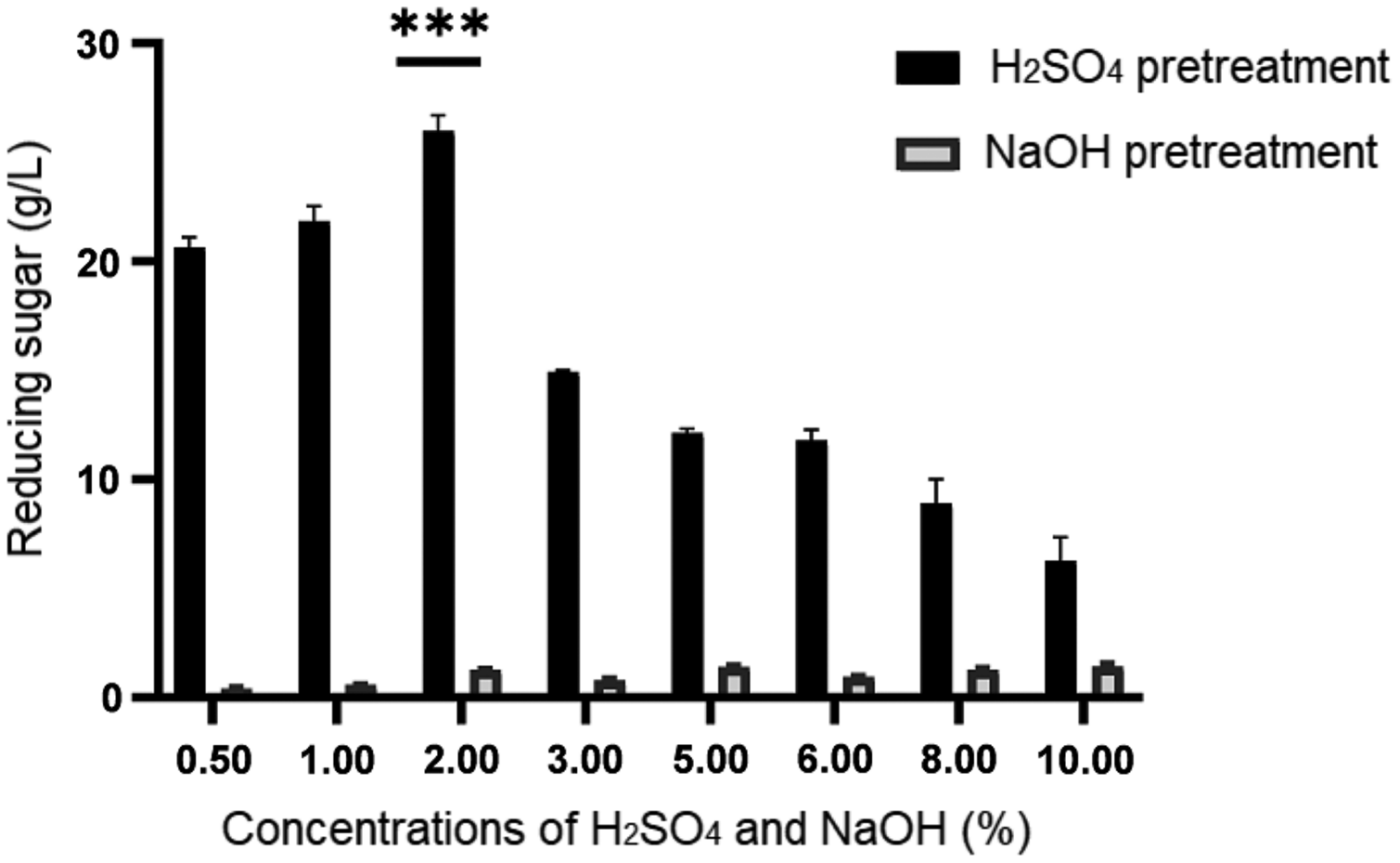
Concentration of reducing sugar generated upon sulphuric acid (H_2_SO_4_) and sodium hydroxide (NaOH) pretreatment of desiccated coconut residue at concentrations from 0.50% to 10.00%. Data are presented as means ± SE from triplicates. ***P-value < 0.05 (one-way ANOVA with Turkey’s test).

All the treatments were performed in triplicates, and the results were presented as mean ± standard deviation (SD). Data was analyzed by one-way analysis of variance (ANOVA), and the comparison between groups in Fig. 1 was performed using Tukey’s HSD test to determine statistical significance. Statistical analysis was performed using GraphPad Prism version 7.0 software (La Jolla, CA, USA). The significance was set at *p* < 0.05.

## Results

### Effect of acid and alkaline pretreatment on reducing sugar production

By referring to the results shown in Fig. 1, it illustrated a remarkable difference in the concentrations of reducing sugar produced from both pretreatment methods. Generally, the amount of reducing sugar produced from different concentrations of alkaline pretreatment was much lower (below 1.45 g/L) than that of acid pretreatment (>6.28 g/L). Despite its low performance, a higher concentration of reducing sugar ranged from 1.42 ± 0.06 g/L to 1.45 ± 0.12 g/L respectively, was obtained at relatively high concentrations of NaOH (5% to 10%). Among the eight different concentrations of H_2_SO_4_ being tested in this study, it was noticeable that the reducing sugar productions at 0.5%, 1.0%, and 2.0% were higher than the rest, with 2% ranked as the top. Hence, from the result obtained, 2% H_2_SO_4_ was used to treat the desiccated coconut residue for the subsequent procedures prior to fermentation as it could produce the highest reducing sugar (25.98 ± 0.71 g/L).

### Effect of potential inhibitors on cell growth

**Figure 2:**
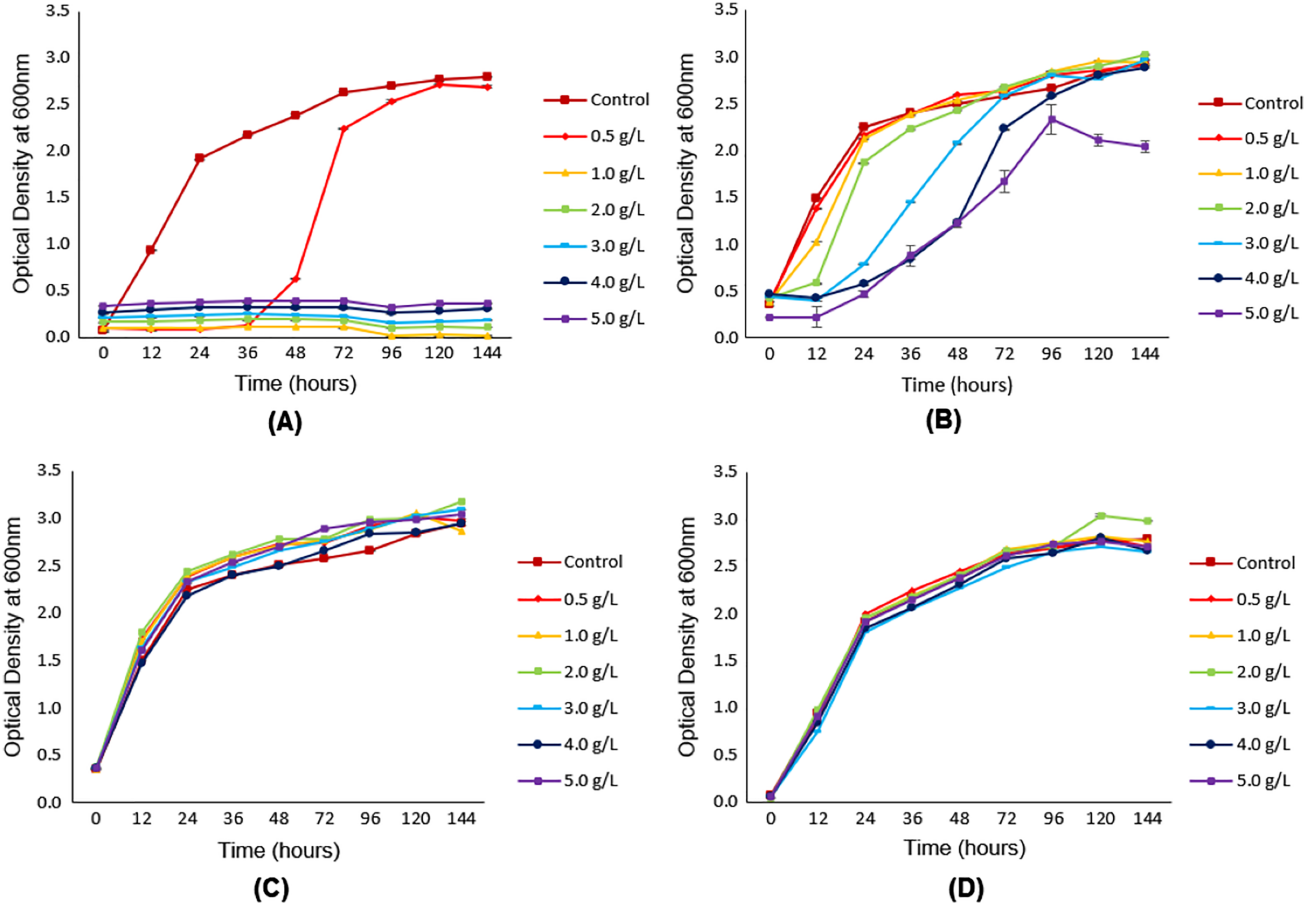
Growth of *Y. lipolytica* JCM 2320 in YPD synthetic media with (from 0.5 g/L to 5.0 g/L) and without (control) the addition of different concentrations of (A) furfural, (B) HMF, (C) acetic acid and (D) levulinic acid. Error bar represented standard deviation derived from the triplicate experiment set.

Figure 2A clearly showed the influence of furfural on the growth of oleaginous yeast *Y. lipolytica* JCM 2320. Compared to the control flask (with the absence of furfural), the lag phase for *Y. lipolytica* JCM 2320 was observed to exist in a longer period, as indicated by a reduction of relative cell growth up to 36 h in the presence of 0.5 g/L furfural. Increasing the concentration of furfural beyond 0.5 g/L was seen as detrimental to the growth of *Y. lipolytica* JCM 2320, as illustrated by the growth profile.

Figure 2B depicted the influence of HMF at various concentrations, ranged from 0.5 g/L to 5.0 g/L, on the growth profile of *Y. lipolytica* JCM 2320. As shown, the relative growth profile of *Y. lipolytica* JCM 2320 with the addition of 0.5 g/L HMF was comparable with the control (with the absence of HMF) flask, showing no inhibitory effect at the lowest HMF concentration. An increase in HMF concentration, starting from 1.0 g/L HMF, was observed to be accompanied by increasing periods for the lag phase. At 96^th^ h, *Y. lipolytica* JCM 2320 cultivated in YPD medium added with 4.0 g/L HMF showed an almost identical growth rate to the control medium. At 5.0 g/L HMF, the relative growth (%) was slightly decreased at 120 and 144 h, confirming that *Y. lipolytica* JCM 2320 could only tolerate up to 4.0 g/L HMF.

As shown in Figs. 2C and 2D, no single inhibitory effect was observed on the growth of *Y. lipolytica* JCM 2320 in the YPD synthetic medium introduced with both acetic and levulinic acid, even at the highest concentration (5.0 g/L). Although acetic acid is well-known for its inhibitory effect on cell growth, however, in this case, it did not cause any inhibitory effects on the growth of *Y. lipolytica* JCM 2320. In contrast, the growth of *Y. lipolytica* JCM 2320 was observed to be slightly higher than the control flask (with the absence of acetic acid), as demonstrated in Fig. 2C.

### Effect of undetoxified hydrolysate on cell growth

**Figure 3:**
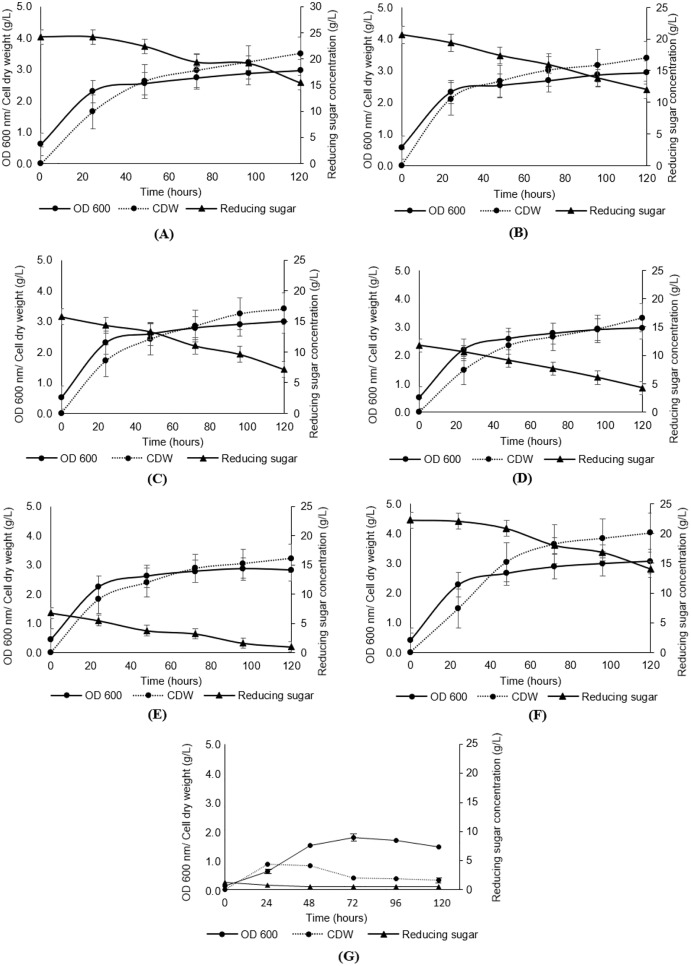
Analyses of growth (OD600), cell dry weight (CDW) and reducing sugar from the cultivation of *Y. lipolytica* JCM 2320 with desiccated coconut hydrolysate media, YPD synthetic medium and YP medium. (A) Hydrolysate with initial 25 g/L sugar (Medium 25); (B) hydrolysate with initial 20 g/L sugar (Medium 20); (C) hydrolysate with initial 15 g/L sugar (Medium 15); (D) hydrolysate with initial 10 g/L sugar (Medium 10); (E) hydrolysate with initial 5 g/L sugar (Medium 5); (F) YPD synthetic medium (Medium YPD); and (G) YP medium without sugar (Medium YP). Error bar represented standard deviation derived from the triplicate experiment set.

As presented in Fig. 3, the growth of *Y. lipolytica* JCM 2320 in media composed of undetoxified desiccated coconut residue hydrolysate (Medium 5, Medium 10, Medium 15, Medium 20 and Medium 25) showed comparable results to that observed for the standard YPD synthetic medium (Medium YPD), neither prolong lag phase nor complete inhibition. In the meantime, the low growth rate of *Y. lipolytica* JCM 2320 observed in a control medium containing yeast extract and peptone without sugar (Medium YP) confirming the role of desiccated coconut residue hydrolysate in supporting the cell growth. Therefore, *Y. lipolytica* JCM 2320 was proposed to have a higher tolerance to the common inhibitors presented in hemicellulose hydrolysate, which was in agreement with the results obtained for the inhibitory study. The cell dry weight showed a sign of increase across the fermentation hours, where the highest cell dry weight was obtained at 120^th^ fermentation hours for all fermentation media, as evidenced in Figs. 3A–3G. As for reducing sugar, it showed a steady reduction in sugar concentration, and residual sugar was observed in all fermentation media. For instance, *Y. lipolytica* JCM 2320 cultivated in Medium YPD utilized about 8.29 g/L of glucose which was comparable to the data obtained in Medium 25 to Medium 5 (with respective quantified sugar from hydrolysate of 25, 20, 15, 10 and 5 g/L), with the utilization of sugar in the range of 6.78 to 8.74 g/L.

### Effect of undetoxified hydrolysate on lipid content

**Figure 4:**
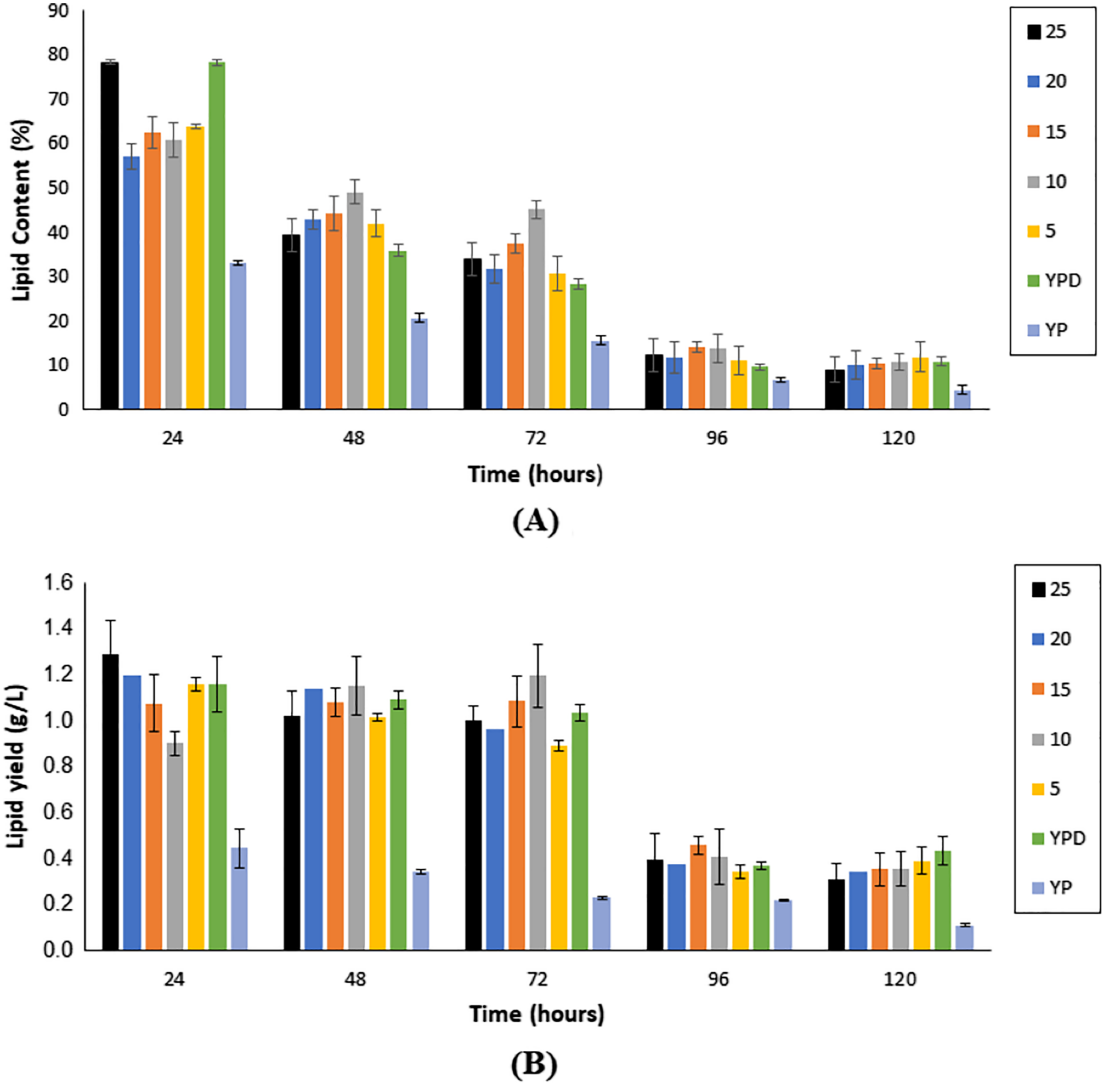
Lipid content (A) and single cell oil yield (B) accumulated upon the cultivation of *Y. lipolytica* JCM 2320 on different media for 120 h fermentation. Error bar represented standard deviation derived from the triplicate experiment set.

As illustrated in Fig. 4A, lipid content showed a reduction with an increase in fermentation hours, in all fermentation media, with the maximum lipid content attained at the 24^th^ h for all media. Nonetheless, Medium 25 (hydrolysate medium with an initial sugar of 25 g/L) reported the highest lipid content, with a value of 78.30 ± 0.57%, followed by Medium F (YPD synthetic medium) at 78.28 ± 0.69%.

As illustrated in Fig. 4B, the yield of SCO accumulated in the first 72 h of fermentation was generally higher if compared to prolong period of up to 120 h. Nonetheless, fermentation hours that accumulated the highest yield of SCO varied accordingly to the medium. For Medium 25, Medium 20, Medium 5, Medium YPD and Medium YP, 24 h fermentation generated the highest yield of SCO, whereas, for Medium 15 and Medium 10, it was obtained upon 72 h fermentation (Table 1).

**Table 1:** Maximum values of single cell oil yield (g/L) and hours obtaining maximum yield (h) for *Y. lipolytica* JCM 2320 growing on different media (*N* = 3).

Medium	Maximum yield (g/L)	Hours obtaining maximum yield (h)
Medium YP	0.44 ± 0.09	24
Medium 15	1.08 ± 0.11	72
Medium 5	1.16 ± 0.03	24
Medium YPD	1.16 ± 0.12	24
Medium 10	1.20 ± 0.14	72
Medium 20	1.20 ± 0.10	24
Medium 25	1.29 ± 0.14	24

**Note:**

Results presented were the mean of three replications ± standard deviations.

### Kinetic parameters

The kinetic parameters of this study are projected in Table 2. Cell dry weight yield coefficient per substrate (YX/S) was obtained within the range of 0.39 to 0.49 for six different media utilized. The highest Y_P/X_ was obtained on Medium A, with a value of 0.37. Lastly, in terms of SCO yield coefficient per substrate (Y_P/S_), values were obtained in the range of 0.13 to 0.17, respectively.

**Table 2:** Overall kinetic parameters of *Y. lipolytica* JCM 2320 upon its cultivation in different fermentation media for 120 h.

Media	Y_X/S_	Y_P/X_	Y_P/S_
Medium 25	0.40	0.37	0.15
Medium 20	0.39	0.35	0.14
Medium 15	0.39	0.32	0.13
Medium 10	0.39	0.36	0.16
Medium 5	0.48	0.36	0.17
Medium YPD	0.49	0.29	0.14

### Effect of detoxified hydrolysate on SCO production

**Figure 5:**
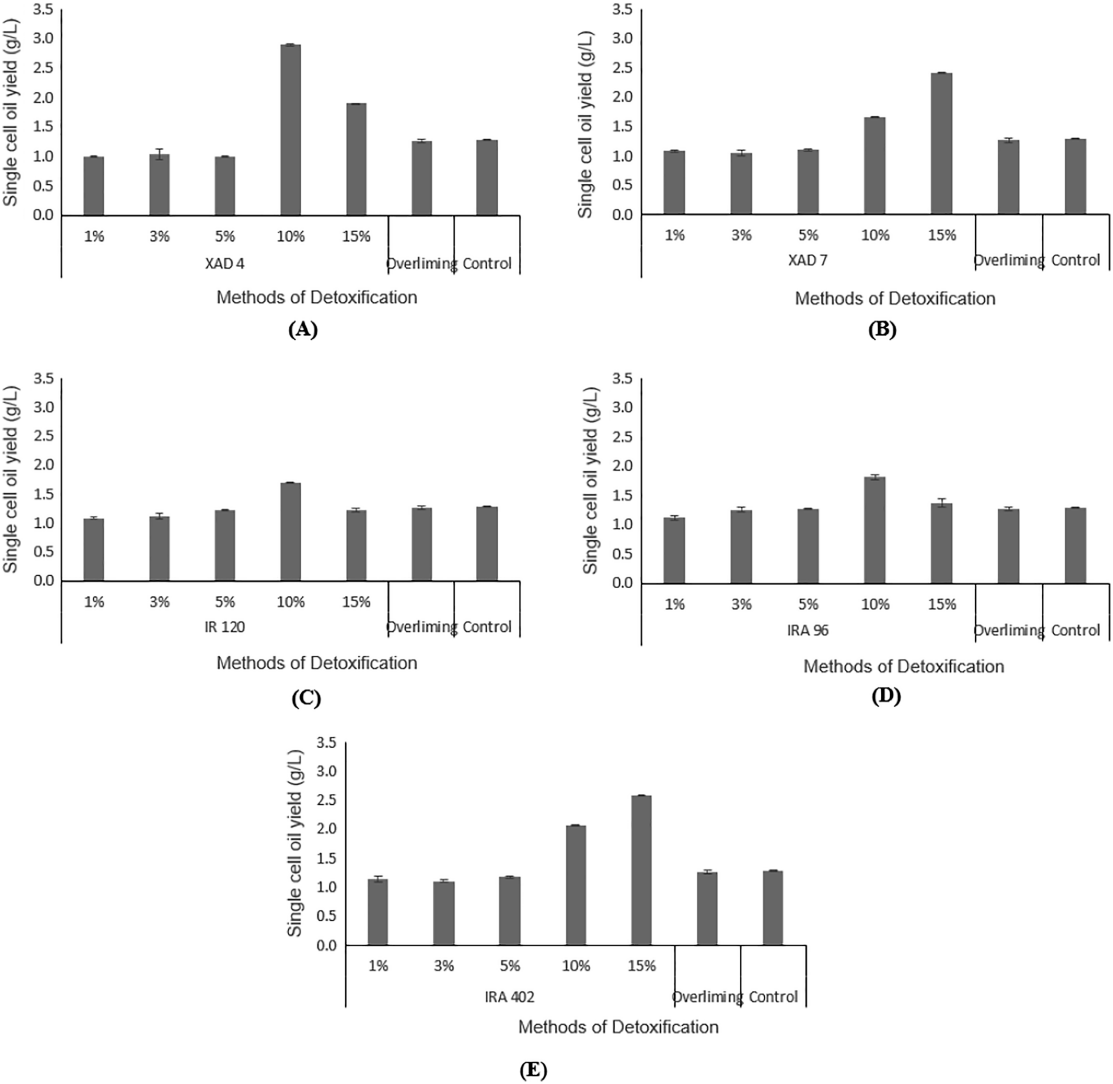
Comparison on single cell oil yield from *Y. lipolytica* JCM 2320 cultivating in control medium and hydrolysate media upon overliming and detoxification for 24 h using polymeric adsorbent resins. (A) Amberlite® XAD-4; (B) Amberlite® XAD-7; Cation Exchange Resin which is (C) Amberlite® IR 120; Anion Exchange Resin which is (D) Amberlite® IRA 96 and (E) Amberlite® IRA 402. Error bar represented standard deviation derived from the triplicate experiment set.

Hydrolysate detoxified with overliming method generated SCO yield at a slightly lower concentration (1.27 ± 0.02 g/L) than that of the control flask (without undergoing any detoxification) (1.29 ± 0.01 g/L). According to the findings displayed in Figs. 5A and 5B, hydrolysate detoxified with Amberlite® XAD-4 resulted in a higher SCO value (2.90 ± 0.02 g/L at 10% concentration) than the other polymeric adsorbent, Amberlite® XAD-7 (2.40 ± 0.01 g/L at 15% concentration).

Hydrolysate detoxified with Amberlite® IR 120 in hydrogen form with 1%, 3%, 5%. 10% and 15% accumulated SCOs of 1.09 ± 0.01 g/L, 1.11 ± 0.05 g/L, 1.22 ± 0.11 g/L, 1.70 ± 0.01 g/L, 1.22 ± 0.04 g/L, respectively, which were lower than the control flask, except at 10% concentration (Fig. 5C). Based on the increasing values of accumulated SCOs with increasing resin concentration (from 1% to 10%), it is concluded that 10% is the best concentration for this cation exchange type of resin.

As depicted in Fig. 5D, a weak base anion exchange resin, Amberlite® IRA 96, effectively enhanced its SCO accumulation compared to the control flask (1.29 ± 0.01 g/L). An increase in the resin concentrations from 1% to 10% would increase the SCO accumulation. At 10% concentration, Amberlite® IRA 96 has attained higher SCO accumulation (1.81 ± 0.03 g/L) than that of the overliming method (1.27 ± 0.02 g/L). Likewise, the accumulation of SCO for the strong base anion exchange resin, Amberlite® IRA 402 in chloride form, (which was in the range of 1.14 ± 0.01 g/L to 2.58 ± 0.05 g/L) was also notably higher than that of the overliming detoxification and the control flask (Fig. 5E).

### Fatty acid composition

Table 3 summarizes the fatty acid profile of the SCO produced from *Y. lipolytica* JCM 2320. The lipid fatty acid profile allows the determination of potential applications. From the GC analysis, it was found that the highest fatty acid composition was 33.60 ± 1.31% for oleic acid (C18:1), followed by palmitic acid (C16:0) with 14.90 ± 0.17%, stearic acid (C18:0) with 11.20 ± 1.33%, and linoleic acid (C18:2) with 9.90 ± 0.74%. The lowest fatty acid compositions were myristic acid (C14:0) and arachidic acid (C20:0) with 5.0 ± 0.01% and 0.90 ± 0.07%, respectively.

**Table 3:** Fatty acid composition of lipids accumulated in *Y. lipolytica* JCM 2320 growing on desiccated coconut residue hydrolysate.

Fatty acid composition (%)						
Myristic acid C14:0	Palmitic acid C16:0	Margaric acid C17:0	Strearic acid C18:0	Oleic acid C18:1	Linoleic acid C18:2	Arachidic acid C20:0
5.0 ± 0.01	14.90 ± 0.17	0.60 ± 0.07	11.20 ± 1.33	33.60 ± 1.31	9.90 ± 0.74	0.90 ± 0.07

**Note:**

Results presented were the mean of three replications ± standard deviations.

## Discussion

This study chose acid and alkaline pretreatment to treat the desiccated coconut residue, rather than other pretreatment technology such as physico-chemical pretreatment (steam explosion and liquid hot water treatment) or physical pretreatment that utilized higher energy (Baruah et al., 2018). In addition, acid pretreatment can generate sugar monomers from lignocellulosic biomass without the necessity to introduce any enzymes (Brodeur et al., 2011). For acid pretreatment, H_2_SO_4_ that received the greatest attention and possessed characteristics such as low risks of usage and highly active, was chosen to be used in this study. However, a number of studies stated that dilute H_2_SO_4_ could lead to the formation of a potential inhibitory compound, *i.e*., furfural in higher concentration if compared to that of maleic and fumaric acid (Singh, Suhag & Dhaka, 2015). In terms of alkaline pretreatment, NaOH known as one of the strongest base catalysts, was used. The selection was owing to its best performance in digesting the biomass without generating inhibitory compounds (Kim, Lee & Kim, 2016; Singh, Suhag & Dhaka, 2015).

Compared to alkaline pretreatment, acid pretreatment was more effective in degrading cellulose due to the removal of hemicellulose and a small amount of lignin (Wang et al., 2020). This was in accordance with the results obtained, whereby reducing sugar with concentrations ranged from 6.28 ± 1.06 g/L to 25.98 ± 0.71 g/L were generated upon acid pretreatment. With the presence of hydronium ions in an acid catalyst, the glycosidic bond between hemicellulose and cellulose in lignocellulosic biomass was degraded into sugar monomers, leading to the high concentration of reducing sugar detected (Baruah et al., 2018). However, it was noticeable that the reducing sugar productions were decreasing with the increment of H_2_SO_4_ concentrations at above 2% (Fig. 1). This observation was also reported by Baadhe, Potumarthi & Mekala (2014). They showed that the increasing concentrations of acid released lesser fermentable sugars along with more inhibitors such as furfural and HMF due to sugars degradation. The high concentration of acid is not adequate to hydrolyze the crystalline cellulose which remains as an insoluble solid and consequently causes both the cellulose and lignin fractions almost unaltered in the solid phase. In the meantime, NaOH treatment released a low amount of sugars compared to H_2_SO_4_ and the values were also found fluctuated in between 2% to 10% concentrations of NaOH. While NaOH pretreatments selectively remove lignin, nevertheless at certain concentrations it also degrades the hemicelluloses in large amounts (Sindhu et al., 2014). For instant, the use of high concentrations (6–20% w/w) of alkaline was found unfavourable as it causes cellulose dissolution and hinders lignin removals (Bensah & Mensah, 2013).

The introduction of H_2_SO_4_ as the pretreatment of lignocellulosic biomass would lead to inhibitors present in fermentation, which is unfavourable to the fermentation process (Kumar & Sharma, 2017). As revealed by Kim (2018), it was noticed that there is a generation of inhibitors such as furfural, HMF, acetic acid, levulinic acid, formic acid, and phenolic compounds upon the pretreatment of lignocellulosic biomass. Furfural and HMF are both categorized under furan derivatives, and they are the degraded products of pentose and hexose, respectively, phenolic compounds due to lignin degradation. Whereas for acetic acid, its presence is related to removing the acetyl group from hemicellulose and is different from formic and levulinic acid, which is the degraded product of HMF, though they are all categorized as aliphatic acids (Almeida et al., 2007).

Based on Fig. 2A, as the concentration of furfural increases, no growth was observed in the media. This observation is contradicted to HMF that exhibited a similar growth rate to that of the control flask (Fig. 2B). Starting from 1.0 g/L onwards, the growth of *Y. lipolytica* JCM 2320 was strictly inhibited in the presence of furfural, while for HMF, its lag phase was observed to be longer than that of the reference flask. This indicated that in an equal concentration, the inhibitory effect of furfural was much stronger and remarkable than HMF, as previously stated by Almeida et al. (2007). Furthermore, according to Sitepu et al. (2014b), all the 45 oleaginous yeast strains being screened including *Wickerhamomyces ciferii* UCDFST 04-836, *Candida* aff. *tropicalis* UCDFST 10-1087, *Metschnikowia* cf. *pulcherrima* UCDFST 11-1039, *Schwanniomyces occidentalis* UCDFST 73-1, and *Cyberlindnera jadinii* UCDFST 76-80 could grow well in the presence of 0.5 g/L of HMF, with no inhibitory effect observed. In the meantime, they also reported that only 50% of the total strains tested could survive at the same concentration of furfural. Therefore, it can be concluded that HMF was indeed less toxic than furfural.

In general, the result obtained for Fig. 2C was partly similar to the experimental screening carried out by Sitepu et al. (2014a). They discovered that the two strains of *Y. lipolytica* employed in the study could grow well in the presence of 2.5 g/L acetic acid. According to Fontanille et al. (2012), acetic acid with a concentration below 5.0 g/L was proved to be a reliable carbon source for cultivating *Y. lipolytica* without inducing any inhibitory effects. Thus, an assumption could be made whereby acetic acid was utilized as a substrate by *Y. lipolytica* JCM 2320, and therefore, its growth was further enhanced rather than inhibited. Enhancement of the cell growth by acetic acid was also reported by Mussatto & Roberto (2004), where acetic acid could stimulate the fermentation process, provided that the tolerance range of the fermenting organisms towards acetic acid was not exceeded. To conclude, the presence of acetic acid up to 5.0 g/L would enhance the growth of *Y. lipolytica* JCM 2320 as it was being utilized as a carbon source, and therefore, no inhibitory effect was observed.

Another well-known example of inhibitors that have been commonly described is levulinic acid, which is derived from further degradation of HMF (Liu et al., 2019). However, as depicted in Fig. 2D, even when levulinic acid was added up to 5.0 g/L, the yeast growth was not affected. This result was in agreement with a study previously conducted by Chen et al. (2009). They observed the growth of six different oleaginous yeast strains (*Trichosporon cutaneum* 2.1374, *Lipomyces starkeyi* 2.1608, *Lipomyces starkeyi* 2.1390, *Rhodotorula glutinis* 2.704, *Rhodotorula glutinis* 2.107 and *Rhodosporidium toruloides* 2.1389) were not significantly influenced in the presence of levulinic acid up to 10.00 g/L. Instead, levulinic acid was found to induce the growth of *T. cutaneum* 2.1374. The reason that levulinic acid possessed low permeability across plasma membranes had been believed to rise to its low toxicity compared to other acids such as formic acid (Liu et al., 2019). To sum up, it can be stated that *Y. lipolytica* JCM 2320 is a robust strain that possesses higher tolerance towards levulinic acid.

The results obtained in Fig. 3 showed that desiccated coconut residue hydrolysate as a carbon source in the fermentation of oleaginous microorganisms could lead to the same promising growth brought by synthetic glucose. Moreover, this could be an added advantage as it can help to effectively manage the lignocellulosic biomass, which is produced in an enormous amount annually, rather than disposing of it *via* open burning (Bilal et al., 2020). In terms of lipid production, the data from Figs. 3 and 4A showed the lipid contents derived in this study were generally higher if compared to the data reported by Papanikolaou & Aggelis (2011), but comparable to Carsanba, Papanikolaou & Erten (2018) that utilizing a nonconventional yeast, *Yarrowia lipolytica*. They revealed that genus *Yarrowia* could also reach up to 90% of lipid accumulation within the cells. This shows that yeasts differ in the amount of lipid biosynthesis, even among strains of the same species and hence optimizing their culture conditions are important to accelerate and promote the accumulation of lipids (Sitepu et al., 2014b).

According to Carsanba et al. (2020), it was common to obtain the highest yield of SCO after a 2 to 8 days range of fermentation. In terms of the amount of SCO yield, Medium 25 to Medium 5 (composed of undetoxified hydrolysate media) showed similar or even higher SCO yield than the Medium YPD. Medium 25 ranked the top with 1.29 ± 0.14 g/L, followed by Medium 20 (1.20 ± 0.10 g/L). The lowest SCO yield was obtained upon cultivating *Y. lipolytica* JCM 2320 with Medium 15, with only 1.08 ± 0.49 g/L. This was probably due to the high tolerance of *Y. lipolytica* JCM 2320 towards any potential inhibitors that may be present that otherwise could inhibit the pathway for SCO accumulation. Overall, this is an interesting finding since not all strains can grow on undetoxified lignocellulosic hydrolysates (Sitepu et al., 2014b; Caporusso, Capece & De Bari, 2021). From Table 2, the value obtained for SCO yield per dry cell weight (Y_P/X_) was comparable to the study demonstrated by Katre et al. (2012) and Papanikolaou & Aggelis (2011), whereby the theoretical value for Y_P/S_ should be below 0.20 g/g, and the chances to go beyond 0.22 g/g was considered rare with glucose as the carbon source. In short, Medium 25 was chosen to proceed with the subsequent detoxification study, particularly at the 24^th^ h as it was ranked as top in lipid content, SCO yield, and Y_P/X_, even its Y_X/S_ and Y_P/S_ was slightly lower than the Medium 5.

Prior to resin adsorption, SCO yield was generated by the commonly used alternative in detoxifying hemicellulose hydrolysate, namely overliming. Overliming method is generally known for its effective performance in removing furfural and HMF in previous studies, such as demonstrated by Yu et al. (2011). The performance of overliming was dependent on the type of lignocellulosic biomass utilized for hydrolysate production and the concentration of inhibitors presented within it (Kim et al., 2013). Since the overliming method used in this study produced low concentrations of SCO compared to the control, an alternative method employing adsorption phenomenon by resins was applied. To date, none of the resins had been tested for their suitability and performance in detoxifying desiccated coconut residue hydrolysate. Hence, screening of resins from different categories (polymeric adsorbent and anion/cation exchange resins) were conducted at five different concentrations (1%, 3%, 5%, 10% and 15%).

As illustrated in Figs. 5A and 5B, Amberlite® XAD-4 has resulted in a higher SCO, 2.90 ± 0.02 g/L at 10% concentration compared to Amberlite® XAD-7, 2.41 ± 0.00 g/L at 15% concentration of resins, respectively. Likewise, Sainio, Turku & Heinonen (2011) mentioned that Amberlite® XAD-7, with an acrylic resin matrix, was not as effective as that of Amberlite® XAD-4, which was polyaromatic. Wei et al. (2015) stated that Amberlite® XAD-4 was able to remove HMF in pine autohydrolysate from 2.22 to 0.83 g/L and from 1.51 to 0.56 g/L in sweetgum hydrolysate. Whereas for furfural, a significant reduction was noticeable in pine and sweetgum hydrolysate, registering a magnitude of 0.87 to 0.15 g/L, and 3.17 to 0.66 g/L, respectively. On the other hand, Sandhya et al. (2013) demonstrated that both Amberlite® XAD-4 and XAD-7 managed to attain 100% of HMF removal while for furfural, the removal was in the range of 55–70%. Cation exchange resins are commonly applied in eliminating inhibitory compounds that possess hydrophobicity properties, such as HMF and furfural (Lee, 2017). Sainio, Turku & Heinonen (2011) stated that the cation exchange resins had led to a higher portion of furfural removal than HMF. From Fig. 5C, Amberlite® IR 120 was assumed to produce lipid higher than the control flask, which indirectly proves its detoxification ability, even though according to de Mancilha & Karim (2003), it failed to eliminate any acetic acids. In general, the adsorption by anion exchange resins is considered as one of the best detoxification methods as it could lead to the removal of all three categories of common inhibitors in the fermentation (de Mancilha & Karim, 2003; Lee, 2017; Nilvebrant et al., 2001; Wang et al., 2020). In this study, weak and strong base resins were selected to be explored for their efficiency in detoxifying desiccated coconut residue hydrolysate, with Amberlite® IRA 96 and Amberlite® IRA 402 as respective examples. Amberlite® IRA 96, a type of macroporous resin that existed in a free base form, is now receiving great attention among researchers due to its higher porosity compared to other resins that possessed the same gel-matrix (Wang et al., 2020). This weak base anion exchange resin has been utilized in various applications, not only for recovering of lactic acid from fermentation broth (Bishai et al., 2015), but also for removing inhibitory compounds from pulp mill waste (Llano, Quijorna & Coz, 2017). As presented in Fig. 5D, the results verify the detoxifying potential of Amberlite® IRA 96, with the best results obtained at 10% concentration of resin. However, for strong base anion exchange resin, Amberlite® IRA 402 in chloride form shows a higher SCO yield than the weak base anion exchange resin, Amberlite® IRA 96. This finding is contradicted with the result from de Mancilha & Karim (2003). They reported that weak base anion exchange resins showed 100% inhibitory removal from the corn stover hydrolysate, but it was not applicable for strong base anion exchange resins. In short, in this study, Amberlite® IRA 402 was highly potential to be applied in the detoxification of lignocellulosic hydrolysate and was more efficient than the conventional overliming detoxification method. At 15% concentration, the Amberlite® IRA 402 produced the highest yield of SCO (2.58 ± 0.05 g/L), which was even higher than that of control (1.29 ± 0.01 g/L).

Lipid fatty acid profile or fatty acid composition allows the determination of potential applications. Specifically, in the case of biodiesel, the fatty acid profile (*i.e*., chain length and the presence of saturated and unsaturated fatty acid) has a direct effect on the final product properties such as cetane number (CN), viscosity, density and melting temperature (Niehus et al., 2018). The presence of long-chain saturated fatty acids will generate biodiesel with high CN that can be correlated to the reduced NOx (nitrogen oxides) emissions (Patel et al., 2017). This is one of the target properties of biodiesel to replace petroleum diesel with high NOx emissions (Fang et al., 2008). Studies revealed that the chemical constituents of the used feedstock influenced the properties and quality of biodiesel produced. Earlier, *Y. lipolytica* (that was grown on pretreated wheat straw as fermentation media) has been documented by Yu et al. (2011), with the highest fatty acid composition observed to be oleic acid (C18:1) (55.30%) and linoleic acid (C18:2) (20.90%). Meanwhile, Poli et al. (2014) also reported the highest fatty acid composition in oleic acid (17.40 ± 0.59%) and linoleic acid (4.39 ± 0.46%) when *Y. lipolytica* Q21 was cultivated on glucose and ammonium sulphate media. Nevertheless, only those with high contents of stearic acid (C18:0) and oleic acid are the possible targets of interest for biodiesel production (Meng et al., 2009). As shown in Table 3, the result from this work shows considerably high percentages of stearic acid and oleic acid, which were 11.20% and 33.60%, respectively. The value for stearic acid is comparable to the reported studies by Poli et al. (2014) and Dobrowolski et al. (2019), corresponding to 17.40 ± 0.59% and 10.07 ± 0.67%, respectively. Note that the fatty acid composition in this present study revealed that C16 and C18 values are dominant, which were 14.90 ± 0.17% and 11.20 ± 1.33%, respectively. Thus, these crude lipids could be a promising feedstock for biodiesel (Karatay & Dönmez, 2010).

## Conclusions

This work demonstrates the possibility of using hydrolysates from pretreated desiccated coconut residue as a candidate in replacing the synthetic glucose for lipid production by *Y. lipolytica* JCM 2320. The results obtained in this study clearly stated that the Amberlite® XAD-4 resin recorded an SCO yield of 2.90 ± 0.02 g/L, that was higher than that of the control and overliming, with only 1.29 ± 0.01 g/L and 1.27 ± 0.02 g/L SCO accumulation, respectively. This shows the capability of Amberlite® XAD-4 in detoxifying the hydrolysate and enhanced the SCO production. Based on the fatty acid profile, the application of desiccated coconut residue does not negatively influence; in fact, the SCO attained from *Y. lipolytica* JCM 2320 can potentially be utilized for industrial biotechnology applications such as biodiesel production. Nevertheless, further studies are needed to elucidate this prospect.

## Supplemental Information

10.7717/peerj.12833/supp-1 Supplemental Information 1 Raw Data.

## Additional Information and Declarations

### Data Availability

The following information was supplied regarding data availability: All raw data are available in the Supplemental File.
